# Biomarker Investigation Using Multiple Brain Measures from MRI Through Explainable Artificial Intelligence in Alzheimer’s Disease Classification

**DOI:** 10.3390/bioengineering12010082

**Published:** 2025-01-17

**Authors:** Davide Coluzzi, Valentina Bordin, Massimo W. Rivolta, Igor Fortel, Liang Zhan, Alex Leow, Giuseppe Baselli

**Affiliations:** 1 Dipartimento di Elettronica, Informazione e Bioingegneria, Politecnico di Milano, 20133 Milan, Italy or davide.coluzzi@unimi.it (D.C.); giuseppe.baselli@polimi.it (G.B.); 2 Dipartimento di Informatica, Università degli Studi di Milano, 20133 Milan, Italy; massimo.rivolta@unimi.it; 3 Department of Biomedical Engineering, University of Illinois Chicago, Chicago, IL 60612, USA; ifortel@gmail.com (I.F.); weihliao@uic.edu (A.L.); 4 Department of Electrical and Computer Engineering, University of Pittsburgh, Pittsburgh, PA 15260, USA; liang.zhan@pitt.edu; 5 Department of Psychiatry, University of Illinois Chicago, Chicago, IL 60612, USA; 6 Department of Computer Science, University of Illinois Chicago, Chicago, IL 60612, USA

**Keywords:** explainable artificial intelligence, Alzheimer’s disease, magnetic resonance imaging, structural connectivity, neuroimaging biomarkers

## Abstract

As the leading cause of dementia worldwide, Alzheimer’s Disease (AD) has prompted significant interest in developing Deep Learning (DL) approaches for its classification. However, it currently remains unclear whether these models rely on established biological indicators. This work compares a novel DL model using structural connectivity (namely, BC-GCN-SE adapted from functional connectivity tasks) with an established model using structural magnetic resonance imaging (MRI) scans (namely, ResNet18). Unlike most studies primarily focusing on performance, our work places explainability at the forefront. Specifically, we define a novel Explainable Artificial Intelligence (XAI) metric, based on gradient-weighted class activation mapping. Its aim is quantitatively measuring how effectively these models fare against established AD biomarkers in their decision-making. The XAI assessment was conducted across 132 brain parcels. Results were compared to AD-relevant regions to measure adherence to domain knowledge. Then, differences in explainability patterns between the two models were assessed to explore the insights offered by each piece of data (i.e., MRI vs. connectivity). Classification performance was satisfactory in terms of both the median true positive (ResNet18: 0.817, BC-GCN-SE: 0.703) and true negative rates (ResNet18: 0.816; BC-GCN-SE: 0.738). Statistical tests (*p* < 0.05) and ranking of the 15% most relevant parcels revealed the involvement of target areas: the medial temporal lobe for ResNet18 and the default mode network for BC-GCN-SE. Additionally, our findings suggest that different imaging modalities provide complementary information to DL models. This lays the foundation for bioengineering advancements in developing more comprehensive and trustworthy DL models, potentially enhancing their applicability as diagnostic support tools for neurodegenerative diseases.

## 1. Introduction

As the population continues to age, dementia cases are on the rise, imposing serious public health risks and a huge social and economic burden on several countries [1]. The prevalent cause of dementia is currently represented by Alzheimer’s Disease (AD), a neurodegenerative pathology occurring when nerve cells die in the brain and neuronal connections are lost [2]. This condition is known to affect the elderly population and is often associated with significant disability, increased hospitalization, and higher mortality rates [2,3].

Given its incidence and severity, several research efforts have focused on AD whilst neuroimaging techniques such as Magnetic Resonance Imaging (MRI) have emerged as a critical tool for both the diagnosis and monitoring of the disease progression. Various MRI techniques and sequences provide different pieces of data that elucidate critical information relevant to understanding the disease. Early signs of pathological changes in T1-weighted MRI often involve the Medial Temporal Lobe (MTL), particularly hippocampal and parahippocampal atrophy [4,5]. In addition, the brain cortex, especially the temporal and parietal regions, shows atrophic changes in the early stages of the condition [6]. As AD progresses, there is more extensive cortical thinning and ventricular enlargement [4,7]. Additionally, since dendritic, myelin, and axonal loss usually accompanies morphological changes like atrophy [8], the disease assessment can be complemented by diffusion MRI (dMRI) and brain fiber tracking. dMRI offers significant advantages by providing structural connectivity data in the form of an undirected graph, which reveals abnormalities in AD across various scales [9]. Globally, AD manifests as a disconnection syndrome with long-range connection degeneration and altered overall graph topology [10,11]. At the subgraph level, AD has been proved to affect the Default Mode Network (DMN), a set of brain regions involved in memory processes which are subject to atrophy, amyloid protein deposition, and white matter alterations [9,12,13]. Additionally, dMRI studies highlight altered connectivity in the temporal lobe, contributing to memory impairment [9,14] in certain regions of the MTL which are often reported as part of the DMN [15]. These findings have been further corroborated by functional connectivity analyses, which consistently reveal a decreased communication within the DMN [16]. This particular brain network (i.e., DMN) is, therefore, indicated as an extremely important and replicable hallmark of AD from both a structural and functional connectivity perspective [17].

In recent times, the information provided by the abovementioned neuroimaging techniques has been extensively utilized to identify the presence of the disease. In this context, several Machine Learning (ML) methods based on MRI, functional MRI, related features, or connectivity data [18,19] have been developed to differentiate between AD and healthy controls.

Deep Learning (DL) models have also been extensively applied to 3D brain volumes, 2D brain images, and connectivity data. Regarding the first two data types, the investigation of manifold approaches, conducted throughout the years, has resulted in a well-established area of research. In this context, pre-trained models based on Convolutional Neural Networks (CNNs), such as ResNet18, EfficientNet-B0, and VGG, have been largely employed in neuroimaging analyses, providing state-of-the-art performance for different tasks [20,21,22]. With regard to the connectivity data, other approaches—such as the Graph Neural Networks (GNNs)—have been developed. The use of GNNs has recently risen after a decade of development and advancements [23]. These models, along with CNNs and autoencoders, were initially adapted to process brain networks, specifically from functional connectivity data, and then employed for different tasks including mild cognitive impairment classification [24], brain disorder prediction (e.g., AD, autism) [25], regression tasks, and AD staging [26,27]. Despite the extensive efforts toward the development of GNNs, to the best of our knowledge, no studies have yet compared such DL models employing structural connectivity to well-established approaches employing brain neuroimaging scans. This comparison is crucial for evaluating possible significant advantages over traditional approaches, which require minimal preprocessing compared to the connectome extraction and transformations for GNNs.

Regardless of the largely demonstrated effectiveness of DL algorithms, their widespread adoption within clinical settings is limited due to their known “black box” outline. The latest advancements in Explainable Artificial Intelligence (XAI) techniques aim to bridge the gap between the need for high performance and that of understanding the processes leading to the final classification. In this context, no studies i) systematically verify whether the brain regions leveraged by the DL model align with the established knowledge of AD and ii) employ DL in both brain connectomes and neuroimaging data for casting a new light on the neurodegenerative mechanisms underlying the disease.

Among all the existing XAI approaches, the ones used most in neuroimaging studies are the gradient/feature-based methods. Gradients or hidden feature maps are used to determine the importance of different input features with respect to the model’s final predictions. Some examples are represented by Class Activation Mapping (CAM), Gradient-weighted CAM (Grad-CAM), and guided back propagation [28]. These techniques have been largely employed in studies focusing on structural (alias, anatomical) MRI (sMRI) [20,29], but have only recently been used to interpret the decision of models based on structural connectivity [30,31]. Evaluating the interpretability of emerging Artificial Intelligence (AI) approaches against well-established strategies could be beneficial to assess the reliability of results obtained throughout the former. Furthermore, if we consider these two pieces of data (i.e., sMRI or graphs) as complementary resources to assess neurodegeneration in AD, a comparison of their strength and weaknesses may suggest whether their individual utility could be further enhanced by their integration into a unified and more comprehensive model.

To the best of our knowledge, a similar analysis is yet to be conducted. Therefore, in an attempt to fill this gap, we adapt a BC-GCN-SE model originally developed for functional connectivity tasks to structural connectivity data. We implement this novel approach—using brain connectomes—alongside a well-established ResNet18 model using 3D T1-weighted MRI volumes, both leveraging data from the third release of the Open Access Series of Imaging Studies (OASIS-3) [32]. Our primary goals are: (i) comparing the accuracy of these models in classifying AD and (ii) employing Grad-CAM to design an explainability approach able to investigate which data type and model captures features most aligned with known neuroimaging biomarkers of AD. By analyzing and comparing the consistent and divergent features highlighted by each model, we seek to evaluate their specific advantages and to investigate the potential complementarity of T1-weighted volumes and brain connectomes for developing superior and more trustworthy DL models to identify AD.

## 2. Materials and Methods

The methodology is organized into multiple interconnected parts. The first part (Section 2.1 and Section 2.2) addresses data preparation: the selection of the study participants from the OASIS-3 dataset according to specific inclusion criteria, followed by comprehensive data processing steps for both T1-weighted scans and structural connectivity matrixes. The second part focuses on AD classification (Section 2.3, Section 2.4 and Section 2.5), beginning with the training and validation framework, which includes dataset partitioning strategies and methods to address class imbalance issues. Then, this part details two complementary DL approaches: the ResNet18 model, optimized for analyzing 3D T1-weighted volumes, and the BC-GCN-SE model, specifically designed for processing structural connectivity graphs. The final part presents the explainability assessment framework (Section 2.6, Section 2.7 and Section 2.8), which consists of three main components: the implementation of the Grad-CAM technique for visualizing model decisions, the development of a novel relevance measure (based on Grad-CAM) for quantifying regional contributions, and a comprehensive statistical analysis to evaluate whether the brain regions identified as relevant by the models align with known anatomical biomarkers of AD.

### 2.1. Study Population

The dataset used in this study was OASIS-3: a longitudinal collection of data focused on the effects of normal aging and early-stage AD [32]. The dataset, released in 2019, includes 1098 participants among which 605 are cognitively normal adults (i.e., Healthy Controls—HC) and 493 subjects are at various stages of cognitive decline. Each participant underwent both neuroimaging and clinical assessments, both of which were conducted independently throughout the study.

From OASIS-3, the final dataset used for the study was obtained with the following steps: (i) matching the MRI and clinical data by selecting the closest records within a 3-month time span, (ii) matching the dataset of Amodeo and colleagues [33], who extracted the structural connectivity matrixes from Diffusion Tensor Imaging (DTI) data of the original OASIS-3 dataset. This resulting dataset was composed of 692 sessions (age range 42–95 years, mean age 70.06 ± 8.85 years, F:M = 388:304) relative to 543 participants. Each session was associated to a T1-weighted scan and a structural connectivity matrix.

Patients at each session were, finally, differentiated in HC and AD according to the Clinical Dementia Rating (CDR) scale (available in the dataset). The HC class comprised individuals with normal cognitive functions (CDR = 0). The AD class encompassed a spectrum of disease severity, including very mild impairment (CDR = 0.5), mild impairment (CDR = 1), and moderate dementia (CDR = 2). The same subject could have been assigned to both classes, because different CDRs were quantified from different imaging sessions. These data (2.76% of the overall subjects) were retained within the dataset but properly handled during the training/validation process of the DL models (see Section 2.3 for further details). The final ratio between HC and AD sessions was 557:135, with a patient ratio of 428:127 (since 12 subjects displayed a CDR change throughout the study, they were inserted in both classes when dividing the AD subjects from the HC ones; for this reason, the final AD and HC patient sum is equal to 555 instead of the actual 543 subjects).

### 2.2. Data Acquisition and Data Processing

Of all the available T1-weighted scans, 135 were acquired with a 3T Siemens Biograph_mMR scanner, while the remaining 557 with a pair of 3T Siemens TimTrio scanners (Siemens, Erlangen, Germany). Three different imaging sequences were used, as detailed in Table 1.

**Figure 1:**
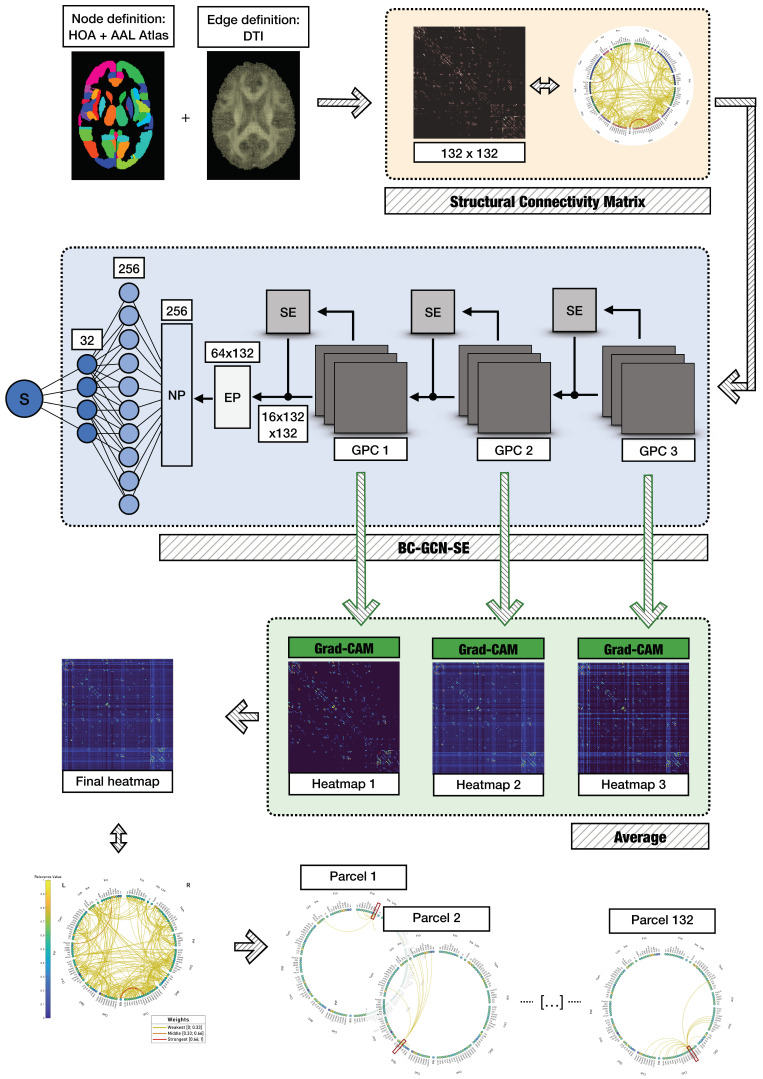
Implemented workflow for connectivity data extraction, AD classification, and explainability assessment performed on BC-GCN-SE. The processing steps used to derive the structural connectivity data, displayed in the orange panel, are described. The architecture of the implemented model (blue panel) comprised three GPC layers, an EP layer, an NP layer, and two fully connected layers. The outputs derived from the three convolutional layers of the model were processed using Grad-CAM (green panel) and then averaged. From the final heatmap, the contributions highlighted by each connection of each node of the HOA + AAL atlas were averaged to extract the Grad-CAM-based RV measure of each parcel. A connectogram representing both connectivity edges of the heatmap and color-coded Grad-CAM-based RV measures (circle perimeter) is displayed. These plots were created using the SPIDER-NET tool (i.e., Software Package Ideal (v1.0) for Deriving Enhanced Representations of brain NETworks) [36].

In a previous study, Amodeo and colleagues [33] processed the imaging dataset to derive the structural connectivity matrixes, which were subsequently used in the present analysis to feed the BC-GCN-SE DL model (see Section 2.5 for further details). To extract the connectivity matrixes, Amodeo and colleagues [33] followed these steps: (i) in the T1-weighted volumes, 132 gray matter parcels were localized—of these, 91 cortical and 15 subcortical parcels were derived from the FMRIB Software Library (FSL, v.6.0) Harvard–Oxford maximum likelihood cortical atlas (HOA) [34], while 26 cerebellar parcels were derived from the Automated Anatomical Labelling atlas (AAL) [35] (henceforth, the combined HOA and AAL atlas will be referred as HOA + AAL), (ii) the combination of the outlined gray matter regions with the DTI white matter fiber tracking resulted in 692 undirected graphs (i.e., positively weighted connectivity matrixes) that underwent a minimal data processing step according to procedures described in [33]. A diagram summarizing the extraction process for structural connectivity matrixes, as outlined by Amodeo and colleagues [33], is reported at the top of Figure 1 and within the orange panel. The subsequent panels illustrate the adopted AD classification strategy (see Section 2.5 for further details) as well as the performed explainability assessment steps (see Section 2.6 and Section 2.7 for further details).

In the present study, we processed the available T1-weighted scans using the FSL v.6.0 tool [37] to create a suitable dataset for the ResNet18 DL model (see Section 2.4 for further details). First, the images were skull-stripped and the bias field corrected using the fsl_anat script (https://fsl.fmrib.ox.ac.uk/fsl/fslwiki/fsl_anat accessed on 15 January 2023). Then, they were registered to the standard Montreal Neurological Institute template (1 × 1 × 1 mm^2^) by applying the non-linear warp transformation provided by fsl_anat. The images field of view was cropped to a dimension of 148 × 180 × 144 voxels to focus on brain tissues while the intensity values were normalized using variance scaling. Eventually, the dimension was resized to 115 × 144 × 118.

### 2.3. Training–Validation Strategy and Evaluation

After pre-processing, the dataset was split into training and validation using a stratified 10-fold cross-validation strategy ensuring that each subject was included in only one of the folds. This approach allowed us to preserve the ratio between AD and HC sessions and to avoid data leakage. Having imbalanced classes within the dataset, before training the BC-GCN-SE model (see Section 2.5 for further details), we applied the Synthetic Minority Oversampling TEchnique (SMOTE) [38] to the training set. This data augmentation method allows to generate synthetic samples through a linear interpolation of real neighboring ones, identified through a k-nearest neighbor approach. This methodology has already been applied to connectivity data in previous studies, though with different target tasks [39,40]. No data augmentation strategy was, instead, carried out before training the ResNet18 model (see Section 2.4 for further details), but the class imbalance issue was dealt with using class weighting.

For both models, the obtained performance was evaluated by calculating, across all 10 folds, the median and interquartile range (IQR) of the True Positive (TPR) and True Negative (TNR) Rates and the median and IQR of the classification accuracy. The cross-validation was performed with an early stopping criterion for validation loss, retaining the model with the highest Area Under the Curve (AUC).

Once the performance was confirmed to be satisfactory through the k-fold cross-validation, the two final models were trained by performing a new split of the original dataset while maintaining the same proportion of the cross-validation step (i.e., 90% and 10% of the entire dataset; the 10% of the dataset was only used to apply the early stopping criterion to the training process) and the same hyperparameters. This configuration was used to derive the final AD/HC classification for the entire dataset. The resulting models went then through XAI to assess the most relevant input contributions (see Section 2.6 and Section 2.7 for further details).

### 2.4. ResNet18: Deep Learning Model for 3D T1-Weighted Volumes

The recognition of AD from 3D T1-weighted volumes was conducted using a Residual Network with 18 layers (ResNet18)—a pre-trained DL model tuned for the multi-class classification of images of the ImageNet database [41]—that was adapted for 3D inputs [42]. Among the CNN-based models that have demonstrated efficacy in sMRI-based classification tasks, ResNet18 was selected after outperforming other pre-trained architectures in AD recognition [43]. The general structure of the model includes a 3D convolutional layer and four sets of residual blocks, each containing two 3D convolutional layers, with a shortcut connection that bypasses the convolutional layers and adds the input directly to the output of the second convolutional layer.

**Figure 2:**
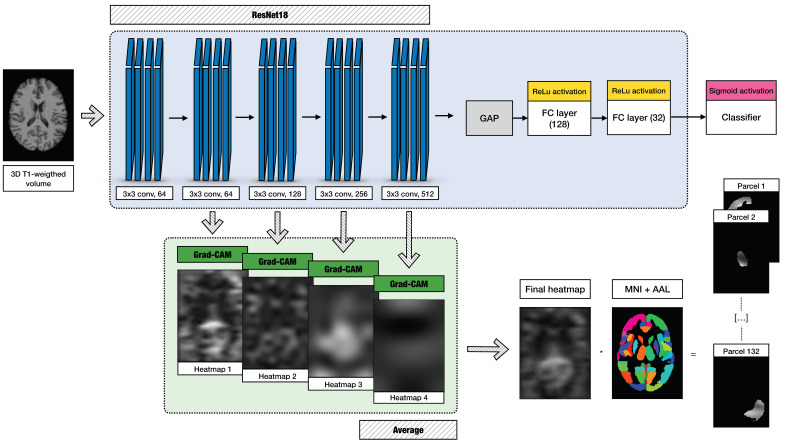
Implemented workflow for the AD classification and explainability assessment performed on ResNet18. The architecture of the implemented model (blue panel) comprised five convolutional layers: a GAP layer, three FC dense layers with ReLU activation, and a sigmoid activation function for the binary classification. The outputs derived from the last four convolutional layers were processed using Grad-CAM (green panel) and then averaged. The final heatmap was then multiplied by the binary masks underlying the HOA + AAL atlas parcels (the operation was indicated using an asterisk).

Transfer learning was employed on the pre-trained ResNet18 by adding to its original architecture a set of layers consisting of a Global Average Pooling (GAP) layer, two fully connected layers (128 and 32 neurons) with Rectified Linear Unit (ReLU) activation, and a sigmoid activation unit. The final network structure is illustrated in the blue panel of Figure 2. The green panel in the same figure shows the explainability assessment steps that are detailed in Section 2.6. and Section 2.7. During the training phase, ResNet18 was optimized using the binary cross-entropy loss function with a batch size of 16. The best model was chosen by applying the early stopping criterion to the validation loss. The model with the highest AUC of the validation set across training epochs was retained.

### 2.5. BC-GCN-SE: Deep Learning Model for Structural Connectivity Graphs

As mentioned in the Introduction, many GNNs—or specifically adapted CNNs for graphs (Graph Convolutional Networks, GCNs)—are often successfully employed in research fields characterized by sparse graphs. However, these methods may not be suitable for the study of brain connectivity data, which are commonly dense or even complete, if severe and arbitrary thresholding is avoided [44]. Among the few models adapted for brain networks, an edge-based GCN—the so-called BC-GCN—has been recently proposed and employed in the functional connectivity field [26]. This architecture has the ability to prioritize graph connections and their weights, rather than relying on explicit node features. This property makes it particularly suited for structural connectivity data obtained through novel DTI tracking methodologies, which bring inherently dense connectomes [44]. The model—that can possibly be extended using a Squeeze-and-Excitation block (BC-GCN-SE)—has shown good performance in both regression and classification tasks involving complete connectivity matrixes [26,27]. For this reason, it was selected and adapted for our AD classification task, using as input the dense structural connectivity graphs. The BC-GCN-SE model was mainly composed of five major units: the Graph Path Convolution (GPC)—which allows for the extraction of feature maps—the Edge Pooling (EP) and Node Pooling (NP) blocks, the Squeeze-and-Excitation (SE) block—to emphasize or suppress feature maps—and the Fully Connected (FC) block used for the final classification.

As said, the communication between different brain areas is achieved through a combination of direct and indirect connections. To extract significant information from these pathways and represent the multi-order information, GPC layers were utilized. This module represents the counterpart of convolutional layers in CNNs, leveraging meaningful characteristics from high-order paths by stacking multiple layers. The EP and NP pooling layers were, instead, employed to aggregate information from nodes and edges, thus downsampling the feature maps which are the output of the convolutional layers. EP and NP are the counterpart of pooling layers in typical CNNs. The SE block was constructed as a typical SE model [26], but the convolution was modified to correspond to the GPC outlined above. The SE layer was positioned after each of the three GPC layers. Finally, the classification part was composed of two FC blocks placed after the NP and before the final sigmoid activation—used to adapt the network to a binary classification task. The resulting BC-GCN-SE model architecture, following hyperparameter tuning, is summarized in the blue panel in Figure 1.

The binary cross-entropy loss function and a batch size of 64 were employed during training. Similarly to the first model, an early stopping procedure was implemented and the model with the highest validation AUC was then retained.

### 2.6. Grad-CAM

To perform XAI on the model’s predictions, we employed a well-established technique called Grad-CAM [45] that generates a heatmap $g\;\in R^{X\;\times \;Y}$ to identify the regions of an input image $i\;\in R^{X\;\times \;Y}$ having the greatest influence on the classifier score in support of a given class c. Grad-CAM’s flexibility allows it to process any CNN architecture, regardless of the application, and obtain relevance heatmaps from any network layer, representing features at different granularities [45]. In the context of our application, the input data (namely, i) varied according to the different DL models employed. For BC-GCN-SE, they represent the structural connectivity matrix, with X and Y being the number of rows/columns (connectivity matrix: $i\;\in R^{X\;\times \;Y\;}$; heatmap: $g\;\in R^{X\;\times \;Y}$) and having equal dimensions (i.e., X = 132, Y = 132). For ResNet18, they represent the 3D T1-weighted volume, with X, Y, and Z being the voxel position (3D scan $i\;\in R^{X\;\times \;Y\;\times \;Z}$; 3D heatmap: $g\;\in R^{X\;\times \;Y\;\times \;Z}$) and having different dimensions (i.e., X = 115, Y = 144, Z = 118).

The heatmap relative to class c is typically achieved by computing a weighted average of the activation maps $a^{k}\in R^{X\;\times \;Y}$ across all the k filters of the selected convolutional layer (k=1, …, K). The summation is followed by a ReLU activation function to consider only the positive contributions:

(1) $$g_{c}=\mathrm{ReLU}\sum_{k=1}^{K}{w_{c}^{k}a}^{k}$$

the $w_{c}^{k}$ values—known as relevance weights—represent the average derivatives of the score for class c ${(s}_{c})$ with respect to each element of the input data in the activation $a^{k}$. Their formulation is reported as follows, for both the 2D and 3D case:

(2) $$w_{c}^{k}=\frac{1}{\mathrm{XY}}\sum_{x=1}^{X}\sum_{y=1}^{Y}\frac{\partial s_{c}}{\partial a^{k}\left(x,y\right)}$$

(3) $$w_{c}^{k}=\frac{1}{\mathrm{XYZ}}\sum_{x=1}^{X}\sum_{y=1}^{Y}\sum_{z=1}^{Z}\frac{\partial s_{c}}{\partial a^{k}\left(x,y,z\right)}$$

### 2.7. Explainability Assessment (Grad-CAM-Based Relevance Value)

Building upon the Grad-CAM technique described in Section 2.6, we developed a novel method to identify the key information utilized by the models.

First, we addressed the issue of heatmap granularity, which is related to the choice of the convolutional layer. Typically, following the implementation of CAM, Grad-CAM is applied to the last convolutional layer. However, this yields an inherent limitation: the resulting heatmap $\left(g_{c}\right)$ exhibits low resolution. This is due to the architecture of the model in which the layer dimensions become more condensed as the network deepens. To facilitate the overlay of the input (i) with Grad-CAM and enhance the interpretation of model decisions, it is thus necessary to upsample the heatmap to match the size of i. Alternatively, to increase the resolution of Grad-CAM, activations from earlier convolutional layers of the network which exhibit higher spatial resolution can be selected. This trade-off between the identification of class-discriminative features with a low spatial extent and fine-grained details has been addressed in several works [46,47,48]. In this work, since we aimed to include it in the contribution to the final classification of features at diverse resolutions and scales (see Section 2.7 for further details) and the last layer alone produced coarse-grained ones due to its inherent architecture, we computed the heatmaps at different layers for every session. For each, we used the bicubic interpolation to match the input size. Then, for every structural connectivity matrix or 3D T1-weighted volume, we averaged such heatmaps across the L available layers:

(4) $$G_{c}\left(x,y\right)=\frac{\sum_{l=1}^{L}g_{c}^{l}\left(x,y\right)}{L}$$

(5) $$G_{c}\left(x,y,z\right)=\frac{\sum_{l=1}^{L}g_{c}^{l}\left(x,y,z\right)}{L}$$

for the two models, the output layers of the four stages dividing ResNet18 and the three GPC-SE blocks in the BC-GCN-SE model were considered, respectively. $G_{c}$ is the resulting mean heatmap.

Second, our approach aimed to assess the relevance of individual brain parcels. This was driven by the need to evaluate which parcels had the greatest impact on the classification process and to facilitate a direct comparison between the level of explainability of the two models. For this analysis, we utilized the combined HOA + AAL atlas of 132 parcels—a choice primarily motivated by its consistency with the atlas used by Amodeo and colleagues [33]—to extract structural connectivity matrixes from the DTI data, further supported by its widespread use in the literature. We began by mapping the obtained mean heatmaps of the AD and HC sessions (see Equations (4) and (5))—generated by both ResNet18 and BC-GCN-SE—onto this atlas. Then, from the mean heatmap of one session, the contribution provided by each connection (located by a pair of rows and columns) of each node (i.e., parcel) of a structural connectivity matrix or by each voxel of each parcel p of a 3D T1-weighted scan was averaged to extract a quantity hereafter called relevance value (RV):

(6) $${\mathrm{RV}}_{p,c\;}=\frac{\sum_{x=1}^{115}\sum_{y=1}^{144}\sum_{z=1}^{118}M_{p}\left(x,\;y,\;z\right)G_{c}\left(x,y,z\right)}{\sum_{x=1}^{115}\sum_{y=1}^{144}\sum_{z=1}^{118}M_{p}\left(x,\;y,\;z\right)}$$

(7) $${\mathrm{RV}}_{p,c}=\frac{\sum_{q=1,\;q\neq p}^{132}G_{c}\left(p,q\right)}{131}$$

where $M_{p}\left(x,\;y,\;z\right)$ is a binary mask obtained from the HOA + AAL atlas to define each parcel p. A diagram summarizing the XAI steps performed on the 3D T1-weighted volumes is reported in the green panel in Figure 2, whereas the structural connectivity data are presented in the green panel in Figure 1.

### 2.8. Statistical Evaluation

The Grad-CAM-based RV measures relative to every HOA + AAL parcel were eventually analyzed as we tried to uncover the processes underlying the classifiers’ decision-making. The analysis was conducted independently for the ResNet18 and BC-GCN-SE models. Additionally, we evaluated, separately, the contribution of each parcel to the classification of AD and HC, considering that the information derived from a single brain parcel could be used differently by the model for detecting the presence or absence of AD. To accomplish this, we initially derived two sets of Grad-CAM-based RV measures for each parcel across the entire population. These sets were created by: (i) removing the misclassified sessions, (ii) averaging the Grad-CAM-based RV measures across single parcels, for subjects with multiple MRI sessions and the same class, (iii) removing the sessions associated with the same subjects but with different classes, and (iv) separating the HC and AD sessions. Subsequently, we applied a novel analysis approach—comprising two different steps—to these refined datasets (see Section 2.8.1).

#### 2.8.1. Statistical Tests and Grad-CAM-Based RV Ranking

To evaluate whether a specific brain region was more relevant for classifying either AD or HC (i.e., if its Grad-CAM-based RV measure was higher for one of the two classes), two statistical tests were carried out. First, the Kolmogorov–Smirnov test was performed on the AD and HC sets relative to every parcel to assess sample normality. Second, the AD and HC sets were compared through the Mann–Whitney or independent samples *t*-test using the Benjamini–Yekutieli correction to account for multiple comparisons [49]. The significance threshold (set to 0.05) thus identified the parcels that were used differently by the algorithms to classify either one or the two classes.

We then shifted our focus to identify the ones that, overall, were most influential for the classification of AD and HC. To this aim, we ranked all parcels based on their Grad-CAM-based RV and selected the 15th percentile having the highest measures for both classes (i.e., 20 out of the 132 total HOA + AAL parcels). The obtained subsets were considered of particular interest for the final classification. From them (i.e., subsets of most relevant parcels for AD and HC), we analyzed two further subgroups that we considered particularly relevant for the classification process: (i) parcels that displayed also a statistically significant difference between the AD and HC Grad-CAM-based RV measures, as these were strongly influencing the model while also providing a greater contribution toward one of the two classes, and (ii) parcels that that did not display a statistically significant difference between the AD and HC Grad-CAM-based RV measures—of these, particular attention was paid to the ones in common between the AD and HC groups, as they were strongly influencing the classification of both classes without leveraging one.

The statistical tests and Grad-CAM-based RV ranking yielded various parcel subsets, which we consistently compared against the established domain knowledge. Specifically, we examined whether these subsets overlapped with brain regions known from the literature to be relevant in AD processes (see Section 2.8.2 for details on these anatomical targets). This comparison allowed us to assess the models’ ability to identify biologically plausible regions, thus being explainable.

#### 2.8.2. Anatomical Targets

Given that the two models utilized distinct types of MRI data (3D T1-weighted volumes and structural connectivity matrixes from DTI), we identified specific anatomical targets for each.

As for the ResNet18 model, a different cluster of parcels was selected as the anatomical target. According to previous studies [8,50], patterns of atrophy in the MTL represent a well-established sMRI biomarker for AD and are often used as a diagnostic criterion for individuals displaying early symptoms. Thus, while conducting the XAI assessment, four structures were considered bilaterally, for a total of eight regions of interest: hippocampus, anterior and posterior parahippocampal gyri, and amygdala.

With regard to BC-GCN-SE, we selected, as the structural connectivity target, a known hallmark of AD: the DMN [9,12,13]. This particular brain network was defined by 31 regions selected according to different previous studies which provided its thorough characterization [51,52,53,54]. The selected regions were then matched with the HOA atlas, using the indications provided in the “Cortical & White Matter Parcellation Manual” (https://cma.mgh.harvard.edu/ accessed on 15 January 2023). Overall, the medial prefrontal cortex, the posterior cingulate cortex, a portion of the temporal lobe, the precuneus, and the inferior parietal areas—like the angular and supramarginal gyri—are often considered part of the DMN. In addition to these core regions, the network also frequently includes the lateral temporal cortex, the hippocampus, and the amygdala [51,52,55]. In this regard, no unique definition exists. While prior research offers preliminary indications of a more comprehensive characterization of the DMN system, additional studies are required to define the anatomical scope of certain parcel contributions [55].

## 3. Results

### 3.1. Classification Performance

The results of the 10-fold cross-validation conducted on the ResNet18 and BC-GCN-SE models are summarized in Table 2.

Both approaches achieved a good performance. Specifically, ResNet18 displayed balanced results for the AD and HC classes (TPR_median_ = 0.817; TPR_IQR_ = 0.073; TNR_median_ = 0.816; TNR_IQR_ = 0.066). The median accuracy was equal to 0.811, while the IQR was equal to 0.073.

BC-GCN-SE achieved an inferior performance compared to the imaging model (TPR_median_ = 0.703; TNR_median_ = 0.738). Furthermore, when comparing the results obtained on the two classes, it provided a slightly better performance and also less variability on the HC during cross-validation (TPR_IQR_ = 0.097; TNR_IQR_ = 0.060). The median accuracy was 0.742, while the IQR reached 0.058.

### 3.2. Explainability Assessment

With regard to the statistical test performed on the Grad-CAM-based RV measures of the AD and HC classes, results relative to all the available parcels are summarized in the Supplementary Materials. Notably, 70 parcels for ResNet18 and 46 parcels for BC-GCN-SE exhibited a significant AD/HC difference (*p* < 0.05). Of these statistically significant parcels, 22 were concordant between the two methods, primarily located in the cortical area—the only exception being represented by two parcels of the cerebellum (see Table S2 for further details).

**Figure 3:**
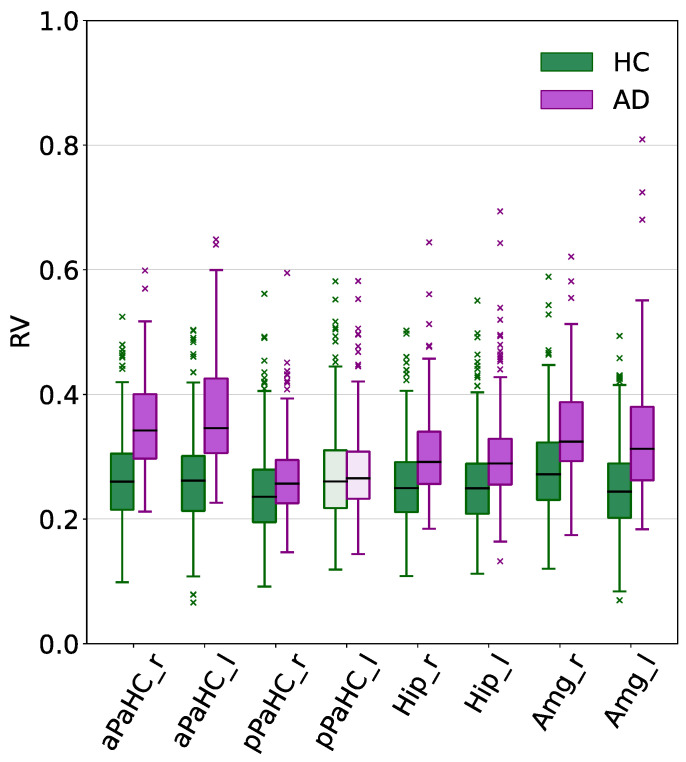
Boxplots of the Grad-CAM-based RV measures relative to the target parcels (specified on the x-axis) of the ResNet18 model. Results for the HC and AD subjects are represented using green and purple, respectively. For visualization purposes, all data were normalized between 0 and 1 using the minimum and the maximum Grad-CAM-based RV measures obtained across every parcel for every subject correctly classified using ResNet18. Lighter shades indicate parcels with significant Grad-CAM-based RV differences between AD and HC groups (Mann–Whitney or independent samples *t*-test).

**Figure 4:**
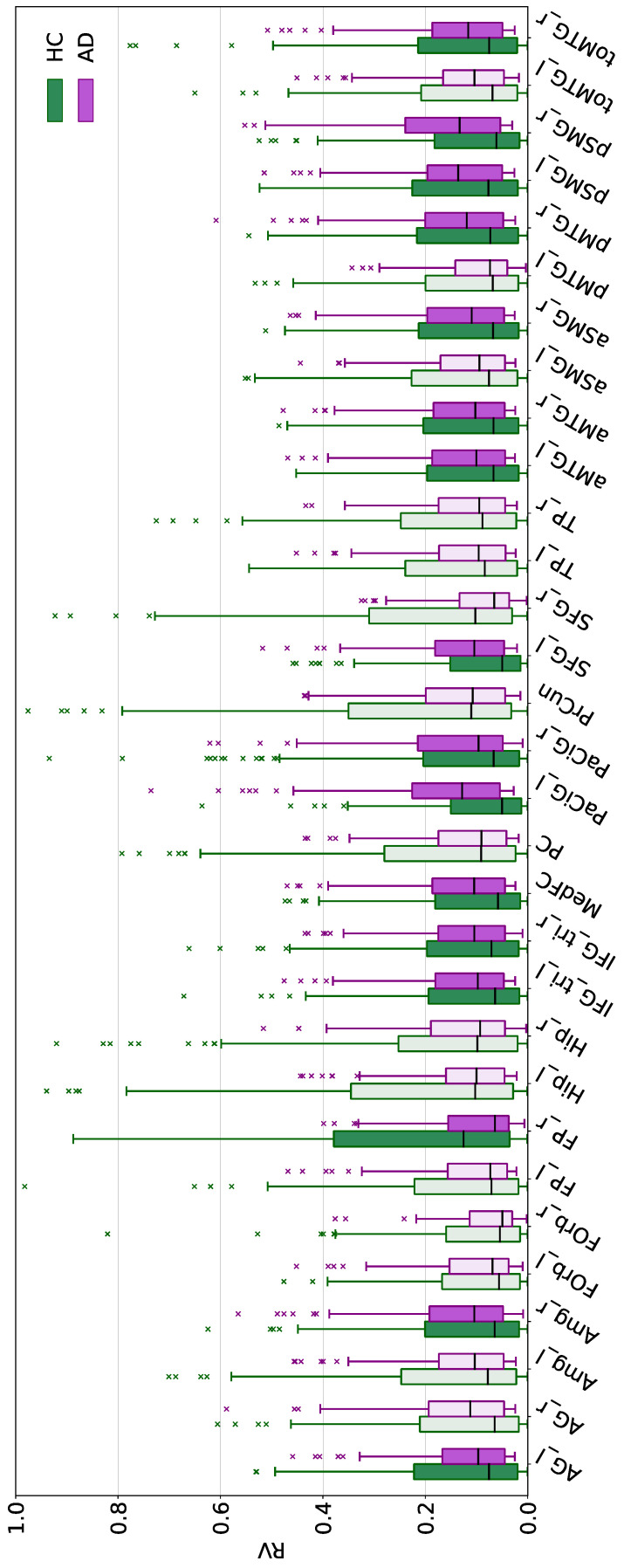
Boxplots of the Grad-CAM-based RV measures relative to the target parcels (specified on the x-axis) of the BC-GCN-SE model. Results for the HC and AD subjects are represented using green and purple, respectively. For visualization purposes, all data were normalized between 0 and 1 using the minimum and the maximum Grad-CAM-based RV measures obtained across every parcel for every subject correctly classified using BC-GCN-SE. Lighter shades indicate parcels with significant Grad-CAM-based RV differences between AD and HC groups (Mann–Whitney or independent samples *t*-test).

Nonetheless, the focus of the present work is represented by target parcels, whose statistical test results are summarized in Table 3. For ResNet18, seven out of the eight total MTL target parcels were found to be significant, with the exception of the posterior division of the left parahippocampal gyrus (i.e., pPaHC_l). Instead, for BC-GCN-SE, all the parcels belonging to the target DMN—except for the frontal orbital cortex (i.e., FOrb_l and FOrb_r), the posterior part of the cingulate gyrus (i.e., PC), the temporal pole (i.e., TP_l and TP_r), the precuneus (i.e., PrCun), and the hippocampus (i.e., Hip_l and Hip_r)—were found to be statistically significant (*p* < 0.05) in at least one hemisphere (12 out of 17 parcels—70.59%). When considering lateralization, the number changed to 16 out of 31 total DMN parcels (51.61%). The distributions of the HC and AD Grad-CAM-based RV measures characterizing the target parcels of both models are displayed in Figure 3 and Figure 4, respectively. The results are visualized as box plots, with anatomical parcels listed along the x-axis and their RV measures—normalized between 0 and 1 for better visualization—on the y-axis. The green boxes represent the HC subjects and the purple boxes represent the AD subjects. Lighter shades indicate parcels where the AD/HC differences did not reach statistical significance (*p* > 0.05).

It is worth noting that most of the target regions that resulted being significantly different, with the exceptions of pPaHC_l for the ResNet18 model and FP_r for the BC-GCN-SE model, showed greater Grad-CAM-based RV measures for the AD case.

Afterward, the most relevant parcels (i.e., highest Grad-CAM-based RV measures) for the classification of AD (see the Supplementary Materials for the full list) were analyzed for both models and compared to the outcomes of the statistical test. First, we noticed that 11 out the 20 most relevant parcels for ResNet18 (Ver10, aTFusC_l, aTFusC_r, aITG_r, SubCalC, aITG_l, aPaHC_l, pITG_r, MedFC, pSTG_l, aPaHC_r) showed statistically significant differences between the AD and HC Grad-CAM-based RV measures. Among them, there were two parcels belonging to the target MTL: the anterior division of both the right and left parahippocampal gyrus. The remaining nine relevant parcels that were not statistically significant were, instead, Cereb10_l, Cereb10_r, Forb_l, Forb_r, CO_r, pSTG_r, PO, Ver12, and Ver3—the first seven of which were also found among the most relevant for the classification of HC subjects.

Secondly, with regard to BC-GCN-SE, 14 out of the 20 most relevant parcels (Cereb1_r, iLOC_r, pMTG_r, pSMG_l, pSMG_r, pSTG_r, toITG_r, ICC_r, OfusG_r, PaCiG_l, PaCiG_r, SPL_r, Tha_l, Ver9) were also characterized by significantly different AD and HC Grad-CAM-based RV measures. Of these, five belonged to the target DMN. The remaining six most relevant parcels that were not statistically significant were, instead, Cereb8_r, Cereb9_l, Cereb9_r, PreCG_r, Ver10, Cereb10_l—the first four of which were also found among the most relevant for the classification of HC subjects.

**Figure 5:**
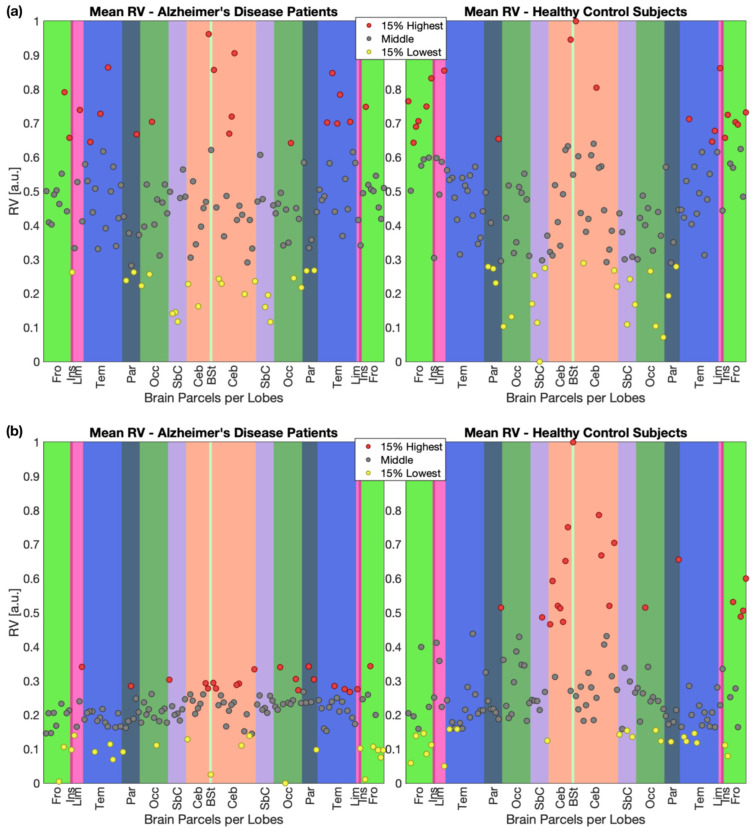
Mean Grad-CAM-based RV measure of all parcels for the ResNet18 (**a**) and BC-GCN-SE (**b**) models. The AD and HC classes are reported separately. For visualization purposes, all data were normalized between 0 and 1 using the minimum and the maximum Grad-CAM-based RV measures obtained across every parcel for every subject correctly classified using the corresponding model. The brain lobe division is indicated through colored rectangles having different size proportionally to the number of parcels contained within each one. The Grad-CAM-based RV measure of every parcel is labeled using colored circles according to the following criterion: red indicates the 15% of parcels characterized by the highest Grad-CAM-based RV measure, yellow indicates the 15% of parcels characterized by the lowest Grad-CAM-based RV measure, gray indicates the remaining parcels. Plots were created using the SPIDER-NET tool [36]. Legend: Fro = frontal lobe; Ins = insular cortex; Lim = limbic lobe; Tem = temporal lobe; Par = parietal lobe; Occ = occipital lobe; SbC = subcortical structures; Ceb = cerebellum; BSt = brainstem.

To allow for the qualitative interpretation of some of the obtained results (see Section 4 for further details), Figure 5 presents a comprehensive visualization of the mean Grad-CAM-based RV measure relative to every parcel, across all the correctly classified sessions, for both ResNet18 (panel a) and BC-GCN-SE (panel b) models. Each panel is divided into two adjacent plots showing AD and HC classes, separately. RV measures, normalized between 0 and 1, are plotted on the y-axis. Along the x-axis, parcels are grouped by brain lobes—each indicated by a distinct colored rectangular background. Within different lobes, parcels are represented by circles, color-coded based on their relative importance: red indicates the top 15% of RV, yellow the bottom 15%, and gray the remaining parcels. This visualization supports the qualitative analysis of which brain regions were most influential in the models’ classification decisions.

Additionally, to allow for a direct comparison between the results obtained by the two models (see Section 4 for further details), a figure displaying the Grad-CAM-based RV measure of the top 15% parcels identified by ResNet18 and BC-GCN-SE for the AD class is included in the Supplementary Materials (Figure S1).

## 4. Discussion

In this work, we compared two AI methods trained on different types of MRI data, namely 3D T1-weighted sMRI scans and structural connectivity matrixes extracted from dMRI data. Both approaches were used for an AD classification task, conducted on a subset of the OASIS-3 data collection. In particular, 543 subjects—comprising both healthy controls and individuals at various stages of the AD cognitive decline—were included in the analysis. The two DL approaches were first evaluated based on model performance (see Section 4.1 for further details), then in terms of explainability (see Section 4.2 and Section 4.3 for further details). Finally, since—to the best of our knowledge—no other AD study has ever conducted a similar investigation, their ability to detect the disease and to utilize well-established brain anatomical targets to generate outcomes were compared (see Section 4.4 for further details). The limitations of the present study are also outlined and discussed in Section 4.5.

### 4.1. DL Model Performance

Overall, the DL models highlighted good classification results. On the one hand, the BC-GCN-SE adapted for structural connectivity achieved a performance comparable to a previous study that proposed and tested a model based on functional connectivity data from the Alzheimer’s Disease Neuroimaging Initiative (ADNI) dataset [25]. Higher specificity (TNR_median_ = 0.738) and slightly lower cross-validation variability (TNR_IQR_ = 0.060) was found for the HC classification compared to the AD cases (TPR_median_ = 0.703; TPR_IQR_ = 0.097). On the other hand, the ResNet18 model, applied to sMRI, achieved a superior performance with respect to BC-GCN-SE when classifying both AD and HC subjects (TPR_median_ = 0.817; TNR_median_ = 0.816). The obtained results were in line with the existing literature [43], while the AD and HC variabilities seemed comparable (TPR_IQR_ = 0.073; TNR_IQR_ = 0.066). To the best of our knowledge, no studies have directly compared classification models using 3D T1-weighted volumes with those employing structural connectivity data. The application of the latter for training DL models is a growing trend that, however, requires careful evaluation against other brain imaging modalities, particularly in terms of processing requirements and classification accuracy. Moreover, this is the first work focusing on the AD classification that tests a BC-GCN-SE model adapted for structural connectivity data derived from the OASIS-3 dataset. Few similar studies have been conducted on different populations, such as the well-known ADNI dataset [25,27], highlighting the good performance of the DL approaches adapted to graph-structured data—mostly functional connectivity.

It is worth noting that connectivity DL models are relatively recent, especially when compared to more mature CNN architectures such as ResNet18. The latter is a widely tested pre-trained model with weights obtained from more than a million images from the ImageNet database, probably resulting in a better ability to generalize. In addition, the structural connectivity data obtained from DTI have inherent limitations related to the processing pipelines that may result in noisy connections. Currently, the absence of gold-standard methods for the creation of connectivity graphs and for edge weighting is indeed one of connectomics’ most severe shortcomings [56,57]. DTI fiber tracking algorithms suffer, for example, from the assumption of a single fiber orientation per voxel, resulting in systematic errors for complex fiber geometries (i.e., crossing, kissing, twisting fibers, etc.). Moreover, since the final data extraction directly depends on the dMRI experiment parameters [56], additional issues may affect the results. While optimal pipelines are yet to be defined, pre-conditioning techniques—such as thresholding methods—are commonly applied to raw connectivity matrixes. These methods may prove beneficial in future research for assessing performance after data cleaning [58].

According to the above-described premises, both DL results can be considered promising. This holds particularly true if we take into account the significant variability characterizing the severities of AD subjects within the OASIS-3 dataset, the presence of different session parameters and of multiple acquisition sessions without a predefined design setting (such as scheduled acquisitions). Furthermore, during the analysis, the subjects’ condition was stratified in two different classes according to the CDR values. This resulted in 12 subjects having sessions with different labels and in a majority of sessions relative to subjects with a very mild impairment being classified as AD (97 sessions with CDR 0.5 out of 135 total AD sessions). Given the broad spectrum of dementia effects and the challenges in precisely defining its stages, this binarization of the target class may have introduced an oversimplification, thus making the final classification more challenging compared to situations in which a more pronounced distinction between classes is present.

As said—despite obtaining satisfying results—the use of brain connectivity graphs for AI is quite recent; therefore, assessing them in comparison to more established approaches using T1-weighted modality would be of great importance. In addition, the possibility of exploring potential biomarkers through explainability methods, in spite of the complex and time-consuming processing that is, sometimes, necessary to extract connectivity data, would cast a new light on the relevant information that could potentially be found in there. This would be of great relevance either for defining the prodromal symptoms of pathologies or to improve diagnosis and rehabilitation.

### 4.2. Explainability Results of the ResNet18 Model

After using the DL models to perform the classification task, we focused on their explainability. The aim was to validate the functioning of AI methodologies employing MRI volumes and structural connectivity matrixes and to assess their agreement with respect to domain knowledge. A Grad-CAM-based RV measure was established to compare the explainability of the classification models across the 132 parcels of the HOA + AAL atlas (see Section 2.7). Then, as outlined in Section 2.8, we compared the Grad-CAM-based RV of each parcel in both the AD and HC subjects by means of statistical tests. Additionally, we extracted the most influential parcels for the classification of AD.

As for the imaging classification task (involving 3D T1-weighted volumes), we saw a significant difference between AD and HC in 70 brain areas. Among these, particular attention was paid to the regions deemed to be more relevant to identify neurodegenerative processes from sMRI data, as indicated by the existing literature. Numerous studies have highlighted the involvement of the MTL in the pathogenesis of AD, and have pointed at its volumetric loss as an early sign of the disease progression [59,60]. Additionally, MTL atrophy has been recently included among the diagnostic criteria for AD [8]. The requirement for dementia onset—once necessary to classify subjects as AD—was also removed in [8], thus making the MTL a reliable biomarker to detect disease presence even before the disability phase occurs (i.e., dementia onset and progression). As mentioned in the Methods section (see Section 2.8.2 for further details), the brain structures involved in the MTL are the hippocampus, amygdala, and parahippocampal regions, which are all key elements for the episodic and spatial memory [50]. However, among these, the most validated and established sMRI biomarker for AD is the hippocampus. A number of studies have linked its volumetric loss to the memory decline stages [4,5,61]. Additionally, Hall and colleagues proved that the amount of time required by cognitively normal subjects to develop dementia is shorter in the presence of hippocampal atrophy [5]. Based on these considerations, we assessed the Grad-CAM-based RV differences characterizing AD and HC subjects in the following MTL parcels: Hip_r, Hip_l, Amg_r, Amg_l, aPaHC_r, aPaHC_l, pPaHC_r, and pPaHC_l. The presence of a significant difference in seven regions (i.e., all except for pPaHC_l) suggests that the algorithm is leveraging them to perform the classification. Additionally, the higher levels of relevance of the AD class compared to HC in all seven structures (see Figure 3) indicates that the DL algorithm is mainly utilizing them to identify the presence of AD. From this, we may infer that ResNet18 seems to be positively influenced by almost all the anatomical regions that are usually involved in the AD progression process. Such an alignment with established domain knowledge suggests the potential clinical relevance of the implemented model.

Given the large number of parcels with a significant AD vs. HC difference—and to further validate our findings—we investigated which parcels, among the significant ones, were also included in the 20 most relevant (i.e., parcels with the highest Grad-CAM-based RV measures) for the AD case. We identified a total of 11 parcels, two of which belonged to the MTL: aPaHC_r and aPaHC_l. This serves as a strong confirmation that the algorithm is leveraging them to identify the presence of AD. Additionally, it is noticeable that almost all the remaining regions (among the most relevant and with a significant AD/HC difference)—the only exception being represented by Vermis10, a parcel belonging to the cerebellum—are part of the brain cerebral cortex, which is known to be involved in the process of disease progression [60]. In particular, six parcels belonged to the temporal lobe (see Figure 5, dark blue rectangles), whose atrophy pattern has been linked to neuronal loss and visuospatial, language, and behavioral impairment by a number of studies [8,62,63]. Furthermore, the inclusion of MedFC and SubCalC—respectively, frontal (see Figure 5, light green rectangles) and limbic cortex (see Figure 5, light pink rectangles)—among the most relevant features aligns with the disease progression pattern described by Eskildsen et al. [4], where atrophy eventually affects most cortical areas except for the visual and sensory motor cortices. Overall, the obtained results indicate that the designed classification approach is correctly using the structural information provided by the 3D T1-weighted images to recognize diseased individuals.

It is worth noting that nine of the 11 parcels discussed so far were indicated as favoring the sole pathological class detection, thus proving their role as an sMRI biomarker for AD. However, in this regard, it is important to mention that seven among the remaining AD most explainable parcels had very high but comparable Grad-CAM-based RV measures with respect to the HC class (see the Supplementary Materials for the full list of parcels characterized by the highest Grad-CAM-based RV measures for the classification of HC subjects). Among these, we found two cerebellar regions (see Figure 5, salmon rectangles) and five cortical regions—frontal, temporal, or parietal (see Figure 5, light green, dark blue and army green rectangles, respectively). As for the cortical regions, we may speculate that the DL model is using them to extract opposite information with respect to the ones generally used to identify AD. The dichotomy between atrophic and physiological cortical volume could play a significant role in the AD/HC distinction. This consideration could still hold for the cerebellar parcels, since the volumetric loss of their molecular and granular layers, despite not being among the most established AD biomarkers, has been linked to the pathology presence by different studies [64,65]. However, further investigation is certainly needed to confirm these aspects or, more generally, shed light on how these specific regions are used by the DL model to discriminate between healthy and pathological individuals.

### 4.3. Explainability Results of the BC-GCN-SE Model

With regard to the relevance of structural connectivity matrixes and their use within the BC-GCN-SE model to classify AD and HC subjects, the DMN was used as the target. Indeed, according to different studies [9,13,66], changes in this brain network are well-known markers of cognitive deterioration, since the Alzheimer’s pathology can alter the WM and disrupt the normal DMN functioning. A recent DTI study has also found that core parcels of the DMN, such as the cingulate cortex and the hippocampus, are gaining even more attention in the AD field since they are also recognized as key structures of the memory system [9]. In the present study, we assessed the relevance of these target regions in the AD classification task through a BC-GCN-SE model (see Section 2.5 for further details), as previously done for ResNet18.

The Grad-CAM result assessment highlighted 46 parcels which contributed differently to the classification of AD and HC subjects (*p* < 0.05). In this regard, we found that more than half of the total parcels belonging to the DMN were among those with significantly higher Grad-CAM-based RV for the AD case. It is also worth noting that an even higher percentage of the target parcels (i.e., more than 70%) was relevantly used by the model if not considering lateralization. In this regard, we acknowledge that there are considerable discussions among researchers and increasing evidence as to the abnormality of topological asymmetry between the hemispheric brain WM in AD and mild cognitive impairment [67,68]. More specifically, all parcels which were found to exhibit asymmetry by our analysis were in agreement with the study by Yang and colleagues [67]. In addition, several of these parcels were also found among the most relevant for the identification of AD. This could serve as a valuable indication—which would be of great interest for further investigation—supporting the presence of hemispheric lateralization and aberration as a result of the long-range connection loss.

Also, the relevance of the changes within the DMN was confirmed by analyzing the statistically significant and—at the same time—most relevant parcels for the AD class. Indeed, among the resulting 14 parcels meeting these criteria, five belonged to the DMN. However, even though, on the one hand, results appeared to indicate a major DMN involvement, on the other, important parcels such as the hippocampus, the posterior cingulate cortex, or the precuneus were not accounted for by the BC-GCN-SE model. The MTL—hippocampus included—also appeared to exert a small influence on the classification process. Apart from the right amygdala, which was found to be statistically significant, other regions such as the parahippocampal cortex appeared to be not relevantly used (*p* ≥ 0.05).

This unexpected pattern might be attributed to several factors. First, the relative novelty of graph-based DL approaches in structural connectivity analyses suggests that these models may not be fully optimized yet to capture established neuroanatomical relationships, possibly focusing on spurious features instead. Additionally, the inherent noise in DTI-derived data—combined with the current lack of standardized processing pipelines—could affect the model’s ability to consistently identify certain anatomical features. This could represent a limitation in terms of explainability for the BC-GCN-SE model, since some of these regions (i.e., hippocampus, parahippocampal cortex) are also often included in the DMN [9], while the MTL has also been found to be relevant in dMRI studies [15]—even though less replicated than the DMN core areas [69]. At the same time, from Figure 5, it is possible to notice that many, among the most relevant parcels, belonged to the right temporal (dark blue rectangles) and parietal (army green rectangles) lobes. In this regard, other studies have highlighted general temporal and parietal lobe disruptions contributing to memory impairment [9,12,66].

Other particular cases examined were represented by the brainstem, nucleus accumbens, and cerebellum, despite not being primary targets. Notably, the brainstem differed significantly between cases, favoring HC, with the highest Grad-CAM-based RV measure, as shown in Figure 5 (central cyan line). At this stage of understanding, we believe that this result may highlight an important influence of the corticospinal tract in healthy individuals [70]. In addition, the brainstem has been found to be associated with the apolipoprotein status, altering radial diffusivity [71,72]. The nucleus accumbens was then found to be statistically different in both hemispheres, in agreement with the alterations found in AD by Nie and colleagues [73]. Moreover, relevant areas common to both classes (see the Supplementary Materials for the full list) included several cerebellar regions (see Figure 5, salmon rectangles), indicating their role in the classification. This supports recent findings of significant cerebellar involvement in AD, as demonstrated by alterations in both structural and functional domains. For example, refs. [74,75] report structural changes in cerebellar white matter, challenging the traditional view that this region remains largely unaffected in AD. Additionally, studies such as [55] highlight the cerebellum’s connections with DMN cortical regions, further supporting its involvement in cognitive processes impaired in AD.

Even though the role of this brain structure warrants further investigation, it is important to consider the complexity of the cerebellum, whose highly folded surface accounts for nearly 80% of the neocortex’s surface area and contains extensive connections to cognitive networks [76]. This intricate architecture, combined with current imaging limitations, may increase susceptibility to noise, particularly in certain cerebellar regions that are more challenging to analyze [77].

The uncertainty of the connectivity data could, therefore, have influenced the findings outlined here, particularly those referring to cerebellar regions where data quality is less robust. These factors highlight the need for further multi-modal investigations, as studies combining structural and functional analyses [17] are beginning to uncover a more comprehensive understanding of cerebellar involvement in AD. Future work could clarify whether these findings reflect true disease-related alterations, reveal novel therapeutic opportunities, or were linked with a spurious correlation not connected with the disease.

### 4.4. Comparing and Combining Explanations

The agreement, peculiarities, and limitations of the two DL models employing sMRI and dMRI data emerge as key findings of the present study. First, 22 regions were found to be significantly used by both models to distinguish the pathological condition. Other matches were found in regions with a high ranking (see Figure S1 of the Supplementary Materials), though not passing the significance test, possibly due to the variability in the population and the limited numerosity of the dataset. This holds particularly true for the BC-GCN-SE model, which was trained from scratch. Altogether, the comparison of the two DL models revealed a certain correspondence, not only in the anatomical target (MTL and DMN respectively), but also in cortical regions outside of these. This aspect was particularly evident for the ResNet18 model, displaying numerous significant parcels in such areas. However, the obtained result was not completely unexpected given the widespread atrophy found in most of the brain cortex and the respective connecting tracts.

In general, the two DL models showed a good agreement with respect to their own targets. Furthermore, it is also noticeable how some important regions of the MTL—such as the amygdala, the parahippocampal gyri, and the hippocampus—which were also found to be significant in the AD dMRI data [15], were not underlined by the XAI analysis of BC-GCN-SE. Indeed, apart from the right amygdala, which was found to be statistically significant, all the other MTL regions were highlighted neither from the statistical test nor from the most relevant parcel analysis. This may indicate a potential limitation in the interpretability of the BC-GCN-SE model or the effect of some noise sources inherently present within the connectivity data.

In general, interpreting DL models that operate on high-dimensional connectomic data presents inherent challenges. The complex architecture of these models incorporate numerous parameters that capture intricate patterns within the data. While these patterns contribute effectively to the classification performance, they may not always map directly to established neurobiological features, thus complicating their interpretation. In this context, multi-modal approaches emerge as a potential solution to this challenge, as they may allow for simpler architectures while maintaining a robust performance focusing on the more salient pieces of data. The complementary information yielded by the two models appears promising in the perspective of developing superior and more trustworthy models. Indeed, the use of well-known hallmarks from multiple MRI measures may offer the opportunity to focus on different information that would be of great value and significance if used concurrently. In this context, only a few studies have considered the combination of morphological features from 3D T1-weighted volumes and interregional properties obtained through structural connectivity data, but this may potentially lead to more accurate results and better interpretations [78]. The integration of these models might be achieved through several different strategies, from simple majority voting to sophisticated multi-branch architectures and fusion networks, while incorporating injecting-based explainability methods that force the network to leverage the current domain knowledge.

### 4.5. Limitations and Perspectives

The present study is not without limitations. The first concerns our approach to the AD classification tasks which was treated as a binary problem. As mentioned previously in the Discussion (see Section 4.1 for further details), given the complex nature of AD and its progressive pattern, this choice might have oversimplified the problem, limiting our ability to identify features that are specifically relevant to different stages of the disease. To address this, future research should incorporate early pathological states, such as mild cognitive impairment, along with various AD severity levels. Such an expanded framework would enable a more comprehensive evaluation of our DL models, providing crucial insights into their explainability and trustworthiness when handling complex, clinically relevant tasks.

The explainability framework also raises some challenges. First, the Grad-CAM-based RV measure may benefit from further validation through comparison with alternative XAI approaches. Examples are perturbation and distillate methods [28], as well as the various versions of the Grad-CAM technique developed throughout the years.

Second, the choice of the convolutional layer from which relevance measures are extracted can affect the granularity of the resulting heatmaps, potentially highlighting features of diverse scales [79]. In this study, we calculated individual heatmaps for each layer and computed their average, achieving a balance between the coarse-grained features detected in deeper layers and the fine-grained features identified in the earlier ones. While effective, this approach represents only one of several possible solutions. Recent advancements in Grad-CAM [46,47,48] offer alternative ways to handle feature granularity, providing comparative insights into how this aspect impacts the model interpretation and classification results.

Another significant limitation concerns our approach to quantifying regional importance. In the present work, we focused on the Grad-CAM-based RV measure, which is computed as the average heatmap intensity across each brain parcel. While this approach facilitated a direct comparison between 3D T1-weighted volumes and structural connectivity data, it presents several limitations. First, the extracted values are inherently dependent on the choice of brain atlas used for the analysis. Even though we selected widely adopted atlases from the literature (i.e., HOA + AAL), using alternative parcellation schemes (e.g., Schaefer [80], Desikan–Killiany [34], etc.—characterized by unique levels of granularity) could yield varying results, potentially affecting the replicability of our findings. This atlas dependency should be carefully considered when interpreting our results or conducting similar analyses. Second, our averaging approach may not adequately account for the intrinsic variability within parcels of different sizes. Specifically, larger parcels might exhibit greater internal heterogeneity in relevance values—which could be masked by the averaging process—while smaller parcels might provide more homogeneous measurements. Future research efforts should address this methodological constraint through the development of XAI approaches specifically designed to enable adequate comparisons between brain regions of varying sizes. Possible examples are represented by: (i) systematic perturbation analyses, where regions of interest are selectively removed from the original data with a volumetric constraint to assess their impact on model predictions, and (ii) data-driven identification of relevant regions—e.g., based on peak heatmap intensities—followed by a comparison with established anatomical targets through spatial overlap indicators. Both alternatives would provide complementary approaches to validate our findings through robust quantitative metrics, fostering a deeper understanding of the models’ decision-making process.

Beyond these methodological considerations, several additional paths warrant further investigation. In the case of structural connectivity, for example, it would be appealing to investigate the most relevant long-range connections and their loss in AD [10,11], as opposed to the brain nodes analyzed in the present study. Furthermore, beyond the analysis of the DMN, the consideration of other resting state networks (such as the frontoparietal network in relation to fronto-temporal dementia, etc.) may provide new insights into the employed models.

Complementary analyses on the WM might also be carried out on the 3D T1-weighted volumes to assess potential differences/similarities with respect to the WM metrics related to structural connectivity data. In this regard, a particular case is represented by white matter hyperintensities that can be seen as important biomarkers of the AD condition [81]. In a previous preliminary study, we confirmed their relevance using a DL model that employs fluid attenuated inversion recovery images, despite the low number of subjects involved in the analysis [21]. A possible future perspective would be to investigate their role with respect to connectivity data as well. For example, it would be interesting to employ tools such as Network Modification (NeMo) to assess the effect exerted by these lesions within the connectivity data structure [82].

Finally, while our models show promising results for AD classification, their validation on OASIS-3 alone limits broader generalizability. Although OASIS-3 is a well-curated dataset with multiple advantages (diverse population demographics, large sample size, multiple scanner types), testing our approaches on external datasets would strengthen their validity and support a potential clinical translation. In this regard, a validation step on the ADNI dataset would be particularly valuable, given the successful results already achieved in this context by DL approaches based on graph-structured data.

## 5. Conclusions

In this work, we focused on a subset of AD and HC subjects from the OASIS-3 dataset to compare two DL models working on data from multimodal MRI acquisitions. Specifically, we employed 3D T1-weighted sMRI volumes (ResNet18) and structural connectivity matrixes (BC-GCN-SE) defined according to the HOA + AAL atlas and to dMRI metrics. In this perspective, we assessed the models according to their performance—which, for both, was in line with state-of-the-art results—and explainability. As for the latter, we employed the Grad-CAM XAI method. Its results pointed to anatomical targets representing imaging biomarkers of the pathology in both sMRI and dMRI. More precisely, the involvement of the MTL was found using ResNet18, whereas the DMN appeared to be important in the decision process conducted by BC-GCN-SE. Even though the expected regions were greatly involved in the classification process, a part of them was missing from the explainability analysis of one of the two models. For example, important areas such as the hippocampus or the parahippocampal gyri were identified by ResNet18, whereas they were excluded by BC-GCN-SE. These results may be influenced by several factors, including the relative novelty of graph-based DL approaches and the inherent noise in DTI-derived data, coupled with the lack of standardized processing pipelines. Interestingly, the BC-GCN-SE model also identified cerebellar regions as relevant. Although unexpected, this finding aligns with recent literature studies linking these areas to the AD condition. These combined observations indicated a certain degree of complementarity and potential mutual improvements in the interpretation provided by the two algorithms. In light of these observations, our work puts emphasis on the potential combination of imaging and connectivity data as a means to create better and more reliable AD models. The availability of multiple MRI modalities gives the opportunity to focus on different information such as the interregional properties found in brain connectivity graphs and the morphological characteristics of regions found in the 3D T1-weighted scans. Their concurrent use, in the context of AD classification, could lead to more accurate and—most importantly—interpretable results. In the long-term, this may help raising the level of confidence and trust laid in AI within clinical contexts, thus promoting their use as reliable tools for both the diagnosis and staging of neurodegenerative conditions.

## Figures and Tables

**Table 1:** Acquisition details for the three sequences involved in our study.

	3T Siemens Biograph_mMR	3T Siemens Biograph_mMR	3T Siemens TimTrio
**Sequence**	T1 (MPRAGE_GRAPPA2)	T1 (MPRAGE isoWU)	T1 (MPRAGE)
**TR (ms)**	2300	2400	2400
**TE (ms)**	2.95	2.13	3.16
**Flip angle (degrees)**	8	8	8
**Voxel size (mm^3^)**	1.20 × 1.05 × 1.05	1.00 × 1.00 × 1.00	1.00 × 1.00 × 1.00
**FOV (mm^2^)**	176 × 240	176 × 256	176 × 256
**Slices per slab**	256	256	256
**TI (ms)**	900	1000	1000
**Orientation**	Sagittal	Sagittal	Sagittal
**Number of sessions**	119	16	557

Legend: MPRAGE = Magnetization Prepared Rapid Acquisition Gradient Echo; GRAPPA2 = Generalized Autocalibrating Partially Parallel Acquisition version 2; isoWU = isotropic Weighted Undersampling; TR = repetition time; TE = echo time; FOV = field of view; TI = inversion time. Scanner types (indicated in columns) and their corresponding characteristics (indicated in rows) are highlighted in bold.

**Table 2:** Row-normalized confusion matrix for the ResNet18 and BC-GCN-SE model performance, reported as median values and IQR.

Cross-Validation Normalized Confusion Matrix		Predicted	
**ResNet18**		AD	HC
**Actual**	AD	0.817 [0.773, 0.846]	0.183 [0.154, 0.227]
	HC	0.184 [0.167, 0.233]	0.816 [0.767, 0.833]
**BC-GCN-SE**		AD	HC
**Actual**	AD	0.703 [0.672, 0.769]	0.297 [0.231, 0.328]
	HC	0.261 [0.242, 0.302]	0.739 [0.698, 0.758]

In the confusion matrix, bold font indicates the models used, the ground truth, and the predictions.

**Table 3:** Statistical analysis of differences in Grad-CAM-based RV measures between the AD and HC groups for target brain parcels relative to the ResNet18 and BC-GCN-SE models.

ResNet18 (3D T1-Weighted Volumes)		BC-GCN-SE (Structural Connectivity Matrixes)					
Parcel	Adjust. *p*	Parcel	Adjust. *p*	Parcel	Adjust. *p*	Parcel	Adjust. *p*
aPaHC_r	<0.001 ***	AG_l	0.268	IFG_tri_r	0.044 *	aMTG_r	<0.001 ***
aPaHC_l	<0.001 ***	AG_r	0.025 *	MedFC	0.006 **	aSMG_l	0.464
pPaHC_r	0.009 **	Amg_l	0.508	PC	1.0	aSMG_r	0.031 *
pPaHC_l	1.0	Amg_r	0.017 *	PaCiG_l	<0.001 ***	pMTG_l	1.0
Hip_r	<0.001 ***	FOrb_l	0.124	PaCiG_r	0.020 *	pMTG_r	0.018 *
Hip_l	<0.001 ***	FOrb_r	1.0	PrCun	1.0	pSMG_l	0.032 *
Amg_r	<0.001 ***	FP_l	<0.001 ***	SFG_l	<0.001 ***	pSMG_r	<0.001 ***
Amg_l	<0.001 ***	FP_r	0.018 *	SFG_r	0.073	toMTG_l	0.164
		Hip_l	1.0	TP_l	0.904	toMTG_r	0.044 *
		Hip_r	1.0	TP_r	1.0		
		IFG_tri_l	0.018 *	aMTG_l	<0.001 ***		

Results are relative to the Mann–Whitney or independent samples *t*-tests. They are reported in terms of *p*-value, after performing the Benjamini–Yekutieli correction (* <0.05, ** <0.01, *** <0.001). The full list of acronyms for the HOA + AAL atlas is reported in the Supplementary Materials.

## Data Availability

The code developed in this study will be made available upon reasonable request to the authors.

## Supplementary Materials

The following supporting information can be downloaded at: https://www.mdpi.com/article/10.3390/bioengineering12010082/s1, Table S1: Full list of acronyms; Table S2: Statistical analysis of differences in Grad-CAM based RV measures between the AD and HC groups for all brain parcels relative to the ResNet18 and BC-GCN-SE models; Table S3: List ordered by ranking of the 15% of parcels characterized by the highest RV for the classification of AD and HC subjects obtained using ResNet18 and BC-GCN-SE; Figure S1: Comparative visualization of the Grad-CAM based RV measure for the most relevant brain parcels (top 15% – specified on the x axis) identified by ResNet18 (turquoise bars) and BC-GCN-SE (orange bars) for the AD classification. For visualization purposes all data are normalized between 0 and 1 using the minimum and the maximum Grad-CAM based RV measures obtained across every parcel for every subject correctly classified using the corresponding model.

## Abbreviations

The full list of Abbreviations is reported in Table S1 of the Supplementary Materials.
