# Supplementary material for: Biomarker Investigation Using Multiple Brain Measures from MRI Through Explainable Artificial Intelligence in Alzheimer’s Disease Classification
Source: Bioengineering (Basel). 2025 Jan 17;12(1):82. doi: 10.3390/bioengineering12010082 (PMC11763248; doi:10.3390/bioengineering12010082)
Supplement: Supplementary file 1 [file bioengineering-12-00082-s001.zip › bioengineering-3341809-supplementary.pdf]

Biomarker Investigation using Multiple Brain Measures from Magnetic Resonance Imaging through Explainable Artificial Intelligence in Alzheimer's Disease Classification

SUPPLEMENTARY FIGURES AND TABLES

Table S1. Full list of acronyms.

GENERAL ACRONYMS					
**AAL**	Automated Anatomical Labeling atlas	**FSL**	FMRIB Software Library	**OASIS-3**	Third release of the Open Access Series of Imaging Studies
**AD**	Alzheimer's Disease	**GAP**	Global Average Pooling	**ReLU**	Rectified Linear Unit
**ADNI**	Alzheimer's Disease Neuroimaging Initiative	**GCN**	Graph Convolutional Network	**ResNet18**	Residual Network with 18 layers
**AI**	Artificial Intelligence	**GNN**	Graph Neural Network	**RV**	Relevance Value
**AUC**	Area Under the Curve	**GPC**	Graph Path Convolution	**SE**	Squeeze-and-Excitation
**BC-GCN-SE**	Brain-Connectivity-Graph-Convolutional-Network with Squeeze-and-Excitation	**Grad-CAM**	Gradient-weighted Class Activation Mapping	**SMOTE**	Synthetic Minority Over-sampling Technique
**CAM**	Class Activation Mapping	**GRAPPA**	Generalized Autocalibrating Partially Parallel Acquisition	**sMRI**	structural Magnetic Resonance Imaging

**CDR**	Clinical Dementia Rating	**HC**	Healthy Controls	**SPIDER-NET**	Software Package Ideal for Deriving Enhanced Representations of brain NETworks
**CNN**	Convolutional Neural Network	**HOA**	Harvard-Oxford Atlas	**TE**	Echo Time
**DL**	Deep Learning	**IQR**	Interquartile Range	**TI**	Inversion Time
**DMN**	Default Mode Network	**isoWU**	isotropic Weighted Undersampling	**TNR**	True Negative Rate
**dMRI**	diffusion Magnetic Resonance Imaging	**ML**	Machine Learning	**TPR**	True Positive Rate
**DTI**	Diffusion Tensor Imaging	**MPRAGE**	Magnetization Prepared Rapid Acquisition Gradient Echo	**TR**	Repetition Time
**EP**	Edge Pooling	**MRI**	Magnetic Resonance Imaging	**WM**	White Matter
**FC**	Fully Connected	**NeMo**	Network Modification	**XAI**	Explainable Artificial Intelligence
**FOV**	Field of View	**NP**	Node Pooling		

BRAIN PARCEL ACRONYMS OF THE HARVARD-OXFORD ATLAS COMBINED WITH THE AUTOMATED ANATOMICAL LABELING ATLAS

**AC**	Cingulate Gyrus, anterior division	**HG**	Heschls Gyrus	**PO**	Parietal Operculum Cortex
**AG**	Angular Gyrus	**Hip**	Hippocampus	**PostCG**	Postcentral Gyrus
**aITG**	Inferior Temporal Gyrus, anterior division	**IC**	Insular Cortex	**PP**	Planum Polare
**Amg**	Amygdala	**ICC**	Intracalcarine Cortex	**pPaHC**	Parahippocampal Gyrus, posterior division
**aMTG**	Middle Temporal Gyrus, anterior division	**IFG_oper**	Inferior Frontal Gyrus, pars opercularis	**PreCG**	Precentral Gyrus

**aPaHC**	Parahippocampal Gyrus, anterior division	**iLOC**	Lateral Occipital Cortex, inferior division	**PrCun**	Precuneous Cortex
**aSMG**	Supramarginal Gyrus, anterior division	**LG**	Lingual Gyrus	**pSMG**	Supramarginal Gyrus, posterior division
**aSTG**	Superior Temporal Gyrus, anterior division	**MedFC**	Frontal Medial Cortex	**pSTG**	Superior Temporal Gyrus, posterior division
**aTFusC**	Temporal Fusiform Cortex, anterior division	**MidFG**	Middle Frontal Gyrus	**PT**	Planum Temporale
**BSt**	Brain-Stem	**NAcc**	Accumbens	**Pu**	Putamen
**CaN**	Caudate	**OFusG**	Occipital Fusiform Gyrus	**SCC**	Supracalcarine Cortex
**Cereb1**	Cerebelum Crus1	**OP**	Occipital Pole	**SFG**	Superior Frontal Gyrus
**Cereb2**	Cerebelum Crus2	**PaCiG**	Paracingulate Gyrus	**sIFG_tri**	Inferior Frontal Gyrus, pars triangularis
**Cereb3**	Cerebelum 3	**Pal**	Pallidum	**sLOC**	Lateral Occipital Cortex, superior division
**Cereb45**	Cerebelum 4 5	**PC**	Cingulate Gyrus, posterior division	**SMA**	Juxtapositional Lobule Cortex - formerly Supplementary Motor Cortex
**Cereb6**	Cerebelum 6	**pITG**	Inferior Temporal Gyrus, posterior division	**SPL**	Superior Parietal Lobule
**Cereb7**	Cerebelum 7b	**pMTG**	Middle Temporal Gyrus, posterior division	**SubCalC**	Subcallosal Cortex
**Cereb8**	Cerebelum 8	**Tha**	Thalamus	**TOFusC**	Temporal Occipital Fusiform Cortex
**Cereb9**	Cerebelum 9	**toITG**	Inferior Temporal Gyrus, temporooccipital part	**TP**	Temporal Pole
**Cereb10**	Cerebelum 10	**toMTG**	Middle Temporal Gyrus, temporooccipital part	**Ver12**	Vermis 1 2

**CO**	Central Opercular Cortex	**Ver3**	Vermis 3	**Ver7**	Vermis 7
**Cuneal**	Cuneal Cortex	**Ver45**	Vermis 4 5	**Ver8**	Vermis 8
**FO**	Frontal Operculum Cortex	**Ver6**	Vermis 6	**Ver9**	Vermis 9
**FOrb**	Frontal Orbital Cortex	**pTFusC**	Temporal Fusiform Cortex, posterior division	**Ver10**	Vermis 10
**FP**	Frontal Pole				

Table S2. Statistical analysis of differences in Grad-CAM based RV measures between the AD and HC groups for all brain parcels relative to the ResNet18 and BC-GCN-SE models.

ResNet18 (3D T1-weigthed volumes)				BC-GCN-SE (Structural connectivity matrices)			
**Parcel**	**Adjust. p**	**Parcel**	**Adjust. p**	**Parcel**	**Adjust. p**	**Parcel**	**Adjust. p**
AC	<0.001 ***	AG_l	0.202	AC	1.000	AG_l	0.268
AG_r	<0.001 ***	Amg_l	<0.001 ***	AG_r	0.025 *	Amg_l	0.508
Amg_r	<0.001 ***	aPaHC_l	<0.001 ***	Amg_r	0.017 *	aPaHC_l	0.323
aPaHC_r	<0.001 ***	aSTG_l	1.000	aPaHC_r	0.268	aSTG_l	0.038 *
aSTG_r	0.464	aSMG_l	0.206	aSTG_r	0.073	aSMG_l	0.464
aSMG_r	1.000	aTFusC_l	<0.001 ***	aSMG_r	0.031 *	aTFusC_l	0.415
aTFusC_r	<0.001 ***	BSt	0.024 *	aTFusC_r	0.014 *	BSt	<0.001 ***
CaN_l	<0.001 ***	CaN_r	<0.001 ***	CaN_l	0.291	CaN_r	0.006 **
Cereb1_l	<0.001 ***	Cereb1_r	0.889	Cereb1_l	1.000	Cereb1_r	0.028 *
Cereb2_l	1.000	Cereb2_r	<0.001 ***	Cereb2_l	1.000	Cereb2_r	0.034 *
Cereb3_l	1.000	Cereb3_r	1.000	Cereb3_l	0.909	Cereb3_r	1.000
Cereb45_l	0.227	Cereb45_r	1.000	Cereb45_l	1.000	Cereb45_r	1.000
Cereb6_l	<0.001 ***	Cereb6_r	0.001 **	Cereb6_l	1.000	Cereb6_r	1.000
Cereb7_l	<0.001 ***	Cereb7_r	1.000	Cereb7_l	1.000	Cereb7_r	1.000

Cereb8_l	<0.001 ***	Cereb8_r	<0.001 ***	Cereb8_l	1.000	Cereb8_r	1.000
Cereb9_l	<0.001 ***	Cereb9_r	<0.001 ***	Cereb9_l	1.000	Cereb9_r	1.000
Cereb10_l	1.000	Cereb10_r	0.074	Cereb10_l	0.103	Cereb10_r	0.186
CO_l	1.000	CO_r	1.000	CO_l	1.000	CO_r	1.000
Cuneal_l	<0.001 ***	Cuneal_r	<0.001 ***	Cuneal_l	0.111	Cuneal_r	0.016 *
FO_l	<0.001 ***	FO_r	<0.001 ***	FO_l	0.296	FO_r	0.001 **
FOrb_l	0.756	FOrb_r	1.000	FOrb_l	0.124	FOrb_r	1.000
FP_l	<0.001 ***	FP_r	<0.001 ***	FP_l	0.908	FP_r	0.018 *
HG_l	0.540	HG_r	0.486	HG_l	0.281	HG_r	0.016 *
Hip_l	<0.001 ***	Hip_r	<0.001 ***	Hip_l	1.000	Hip_r	1.000
IC_l	0.122	IC_r	<0.001 ***	IC_l	1.000	IC_r	1.000
ICC_l	1.000	ICC_r	1.000	ICC_l	0.128	ICC_r	0.031 *
IFG_oper_l	<0.001 ***	IFG_oper_r	<0.001 ***	IFG_oper_l	0.142	IFG_oper_r	1.000
IFG_tri_l	<0.001 ***	IFG_tri_r	<0.001 ***	IFG_tri_l	0.018 *	IFG_tri_r	0.044 *
iLOC_l	1.000	iLOC_r	0.094	iLOC_l	0.129	iLOC_r	<0.001 ***
LG_l	1.000	LG_r	1.000	LG_l	1.000	LG_r	0.044 *
MedFC	<0.001 ***	MidFG_l	<0.001 ***	MedFC	0.006 **	MidFG_l	1.000
MidFG_r	<0.001 ***	NAcc_l	<0.001 ***	MidFG_r	0.118	NAcc_l	0.002 **
NAcc_r	1.000	OFusG_l	0.026 *	NAcc_r	0.015 *	OFusG_l	1.000
OFusG_r	1.000	OP_l	<0.001 ***	OFusG_r	0.018 *	OP_l	0.701
OP_r	<0.001 ***	PaCiG_l	<0.001 ***	OP_r	0.741	PaCiG_l	<0.001 ***
PaCiG_r	<0.001 ***	Pal_l	0.006 **	PaCiG_r	0.020 *	Pal_l	0.282
Pal_r	1.000	PC	0.862	Pal_r	0.028 *	PC	1.000
pITG_l	0.006 **	pITG_r	<0.001 ***	pITG_l	0.142	pITG_r	0.111
pMTG_l	0.011 *	pMTG_r	0.079	pMTG_l	1.000	pMTG_r	0.018 *
PO_l	1.000	PO_r	1.000	PO_l	0.034 *	PO_r	0.021 *
PostCG_l	0.003 **	PostCG_r	1.000	PostCG_l	1.000	PostCG_r	0.010 **
PP_l	1.000	PP_r	0.615	PP_l	0.701	PP_r	0.062
PreCG_l	<0.001 ***	PreCG_r	0.024 *	PreCG_l	0.024 *	PreCG_r	1.000
PrCun	<0.001 ***	pPaHC_l	1.000	PrCun	1.000	pPaHC_l	1.000
pPaHC_r	0.009 **	pSMG_l	1.000	pPaHC_r	0.142	pSMG_l	0.032 *
pSMG_r	0.168	pSTG_l	0.010 **	pSMG_r	<0.001 ***	pSTG_l	0.058

pSTG_r	0.480	PT_l	0.112	pSTG_r	0.028 *	PT_l	0.803
PT_r	0.271	pTFusC_l	0.112	PT_r	0.019 *	pTFusC_l	0.753
pTFusC_r	<0.001 ***	Pu_l	1.000	pTFusC_r	0.100	Pu_l	0.516
Pu_r	0.074	SCC_l	0.529	Pu_r	1.000	SCC_l	0.058
SCC_r	1.000	SFG_l	<0.001 ***	SCC_r	0.058	SFG_l	<0.001 ***
SFG_r	0.010 **	sLOC_l	<0.001 ***	SFG_r	0.073	sLOC_l	1.000
sLOC_r	<0.001 ***	SMA_l	0.592	sLOC_r	0.118	SMA_l	0.018 *
SMA_r	0.077	SPL_l	0.427	SMA_r	0.106	SPL_l	1.000
SPL_r	1.000	SubCalC	<0.001 ***	SPL_r	0.018 *	SubCalC	0.105
Tha_l	<0.001 ***	Tha_r	<0.001 ***	Tha_l	0.031 *	Tha_r	0.803
TOFusC_l	<0.001 ***	TOFusC_r	0.128	TOFusC_l	1.000	TOFusC_r	1.000
toITG_l	<0.001 ***	toITG_r	1.000	toITG_l	1.000	toITG_r	<0.001 ***
toMTG_l	1.000	toMTG_r	0.045 *	toMTG_l	0.164	toMTG_r	0.044 *
TP_l	1.000	TP_r	1.000	TP_l	0.904	TP_r	1.000
Ver12	0.306	Ver3	0.516	Ver12	0.044 *	Ver3	0.919
Ver45	0.005 **	Ver6	1.000	Ver45	1.000	Ver6	0.128
Ver7	0.482	Ver8	<0.001 ***	Ver7	0.018 *	Ver8	0.577
Ver9	<0.001 ***	Ver10	<0.001 ***	Ver9	0.024 *	Ver10	0.055
aITG_l	<0.001 ***	aITG_r	<0.001 ***	aITG_l	0.058	aITG_r	0.044 *
aMTG_l	0.038 *	aMTG_r	0.001 **	aMTG_l	0.038 *	aMTG_r	0.042 *

Results are relative to the Mann-Whitney or independent samples t-tests. They are reported in terms of p-value, after performing the Benjamini-Yekutieli correction ($* < 0.05$, $** < 0.01$, $*** < 0.001$). The 22 significant parcels common to both the ResNet18 and BC-GCN-SE models are highlighted in green.

Table S3. List ordered by ranking of the 15% of parcels characterized by the highest RV for the classification of AD and HC subjects obtained using ResNet18 and BC-GCN-SE.

ResNe18		BC-GCN-SE	
AD	HC	AD	HC
Cereb10_l	Ver10	PreCG_r	BSt
Cereb10_r	Cereb10_l	pSMG_r	Cereb9_r
Ver10	PaCiG_r	PaCiG_l	Cereb9_l

aTFusC_l	PaCiG_l	OFusG_r	Cereb2_r
aTFusC_r	MedFC	Cereb1_r	Cereb8_r
FOrb_l	Cereb10_r	ICC_r	PostCG_r
aITG_r	FP_l	SPL_r	Cereb8_l
FOrb_r	FOrb_l	Tha_l	FP_r
SubCalC	FP_r	Ver10	Cereb2_l
aITG_l	FOrb_r	Cereb9_l	PreCG_r
Ver12	CO_r	Cereb8_r	Cereb45_r
CO_r	IFG_oper_l	Cereb9_r	Cereb45_l
aPaHC_l	IFG_oper_r	toITG_r	TOFusC_r
pSTG_r	IFG_tri_r	pSMG_l	PrCun
pITG_r	IFG_tri_l	Ver9	Cereb6_l
PO_l	aSTG_r	Cereb10_l	SFG_r
MedFC	FO_r	PaCiG_r	MidFG_r
Ver3	PO_l	pMTG_r	Hip_l
pSTG_l	MidFG_l	iLOC_r	Cereb7_l
aPaHC_r	pSTG_r	pSTG_r	Cereb1_l

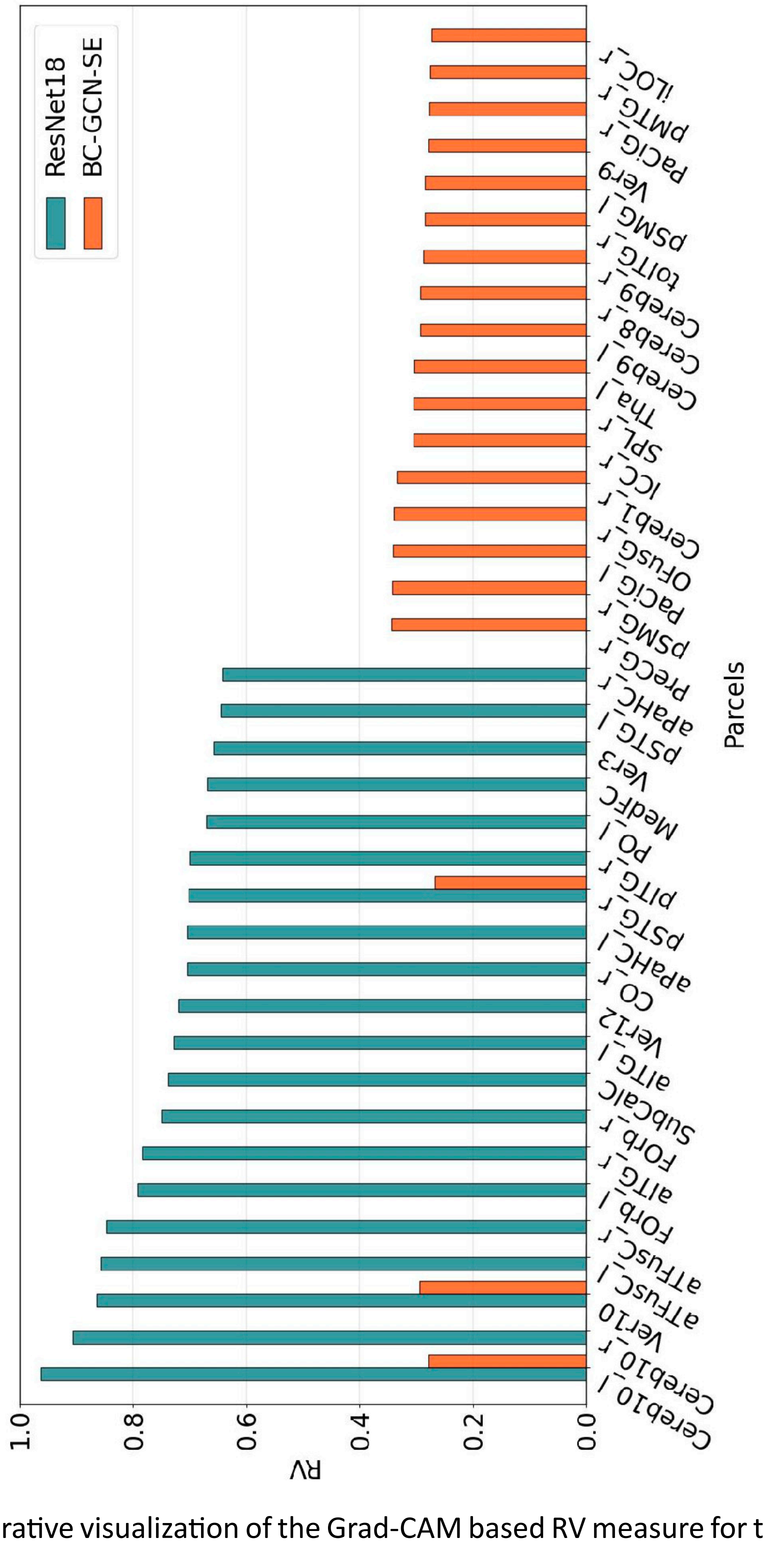

Figure S1. Comparative visualization of the Grad-CAM based RV measure for the most relevant brain parcels (top 15% – specified on the x axis) identified by ResNet18 (turquoise bars) and BC-GCN-SE (orange bars) for the AD classification. For visualization purposes all data are normalized between 0 and 1 using the minimum and the maximum Grad-CAM based RV measures obtained across every parcel for every subject correctly classified using the corresponding model.
